# Did Household Income Loss Have an Immediate Impact on Animal-Source Foods Consumption during the Early Stage of the COVID-19 Pandemic?

**DOI:** 10.3390/foods12071424

**Published:** 2023-03-27

**Authors:** Qi Shen, Taiyang Zhong

**Affiliations:** School of Geography and Ocean Science, Nanjing University, Nanjing 210023, China; qishen1026@163.com

**Keywords:** COVID-19, household income loss, food security, animal-source foods consumption, food affordability

## Abstract

The outbreak of COVID-19 in 2020 caused extensive impact on household income and foods consumption. However, little attention has been paid to the immediate impact of income loss on animal-source foods consumption in the early stage of the COVID-19 pandemic. This paper aims to narrow this gap, and a total of 1301 valid samples of household food consumption surveys in Wuhan and Nanjing were obtained through specially designed online questionnaires. The surveys show that there were 69.6% (Wuhan) and 42.2% (Nanjing) of surveyed households whose animal-source foods consumption were affected, and there were 47.4% (Wuhan) and 18.9% (Nanjing) of surveyed households who suffered income loss. Furthermore, this paper makes an empirical study on the linkage between income loss and animal-source foods consumption. The results show that the pandemic affected household income, resulting in an immediate impact on animal-source foods consumption. This immediate impact may have been due to the combination of price increases, income loss and insufficient savings, which led to a “perfect storm” for animal-source foods consumption. Moreover, household income loss affected various animal-source foods consumption differently. For households suffering income losses, the odds of pork, beef and mutton, poultry, aquatic products, eggs and dairy products consumption being affected were increased by a factor of 1.894, 2.140, 2.773, 2.345, 1.802, 2.835, respectively, holding other variables constant. The results may be related to residents’ consumption habits and food prices. During the COVID-19 pandemic, the reduction of animal-source foods consumption may have led to a state of tension concerning an increase in the development of nutrition intake and health, which may have led to increased food security risks.

## 1. Introduction

The COVID-19 pandemic has caused income loss for some households. COVID-19 is a public health emergency of international concern [1], which has a wide range of impacts [2]. To control the pandemic, many countries have taken a series of lockdown measures, such as strict control of traffic, closure of areas and roads, and prohibition of gatherings. These pandemic containment efforts have effectively controlled the risk of the outbreak and the spread of the pandemic [3,4]. However, the COVID-19 pandemic and mobility restrictions have led to household income loss to some degree. First, the COVID-19 pandemic and its containing measures have caused production interruptions [5], business closures, and unemployment [6], which has directly resulted in income loss in numerous households. Second, firm or business operations have been restricted by policies to contain COVID-19 [6], which may lead to some companies or firms cutting payment (salary or wage) to their employees. The resumption time is prolonged and the working hours reduced [7]. Some employees have had their salaries reduced or have even been laid off [8], resulting in an income impact on people [9,10]. Therefore, these challenges would reduce the income of households whose income source is mainly from wages or salary [8]. A report released by the International Labour Organization on 23 September 2020 estimated that global labor income fell by 10.7%, or USD $3.5 trillion, in the first three quarters of 2020 compared with the same period in 2019 [11]. Some studies have also shown that there was a high proportion of households or individuals that encountered income shock during the COVID-19 pandemic. About 36% of Arkansas adults in the US experienced income loss during the COVID-19 pandemic [12]. The figures for households experiencing income loss were about 9% in Denmark and 23% in Germany [13]. Comparatively, there was a higher percentage of those experiencing income loss in developing countries, such as more than 66% of respondents who experienced income loss in Kenya and Uganda [8].

Income loss could decrease households’ access to food, especially animal-source foods. There have been studies confirming that income loss caused by the COVID-19 pandemic led to an increased risk of food insecurity [12,14]. Animal-source foods are important for household and individual food security. Decreases in animal-source foods consumption lead to food insecurity to some extent, and this has been confirmed in existing research [15]. The reduction in animal-source foods consumption in this paper is mainly due to the limited economic and physical access to animal-source foods. From the economic and physical ACCESS to food dimension of food security, the reduction in animal-source foods consumption increases the risk of food insecurity [16]. Animal-source foods are the main source of natural vitamins, such as vitamin D [17]. Vitamin D is considered important in strengthening the body’s capability of coping with 2019-nCoV infection [18]. In addition, animal-source foods are also rich in protein, with high biological value [19,20], and are more efficient than plant foods in providing essential amino acids demanded by the human body [21]. Protein demand can be higher in those recovering from COVID-19 illness [22]. Therefore, it is important to pay attention to the consumption of animal-source foods to obtain enough high-quality protein, natural vitamins and other nutrients during the COVID-19 epidemic period. A large amount of literature has also shown that animal-source foods play a crucial role in alleviating the risk of undernutrition in growing children and low-income populations in developing countries [23,24]. Faced with income loss, some consumers may shift from expensive food items to less expensive or cheaper categories of foods [25]. Animal-source foods are generally more expensive than plant-based foods, which causes affordability issues with animal-source foods in developing countries in a normal situation [21]. In some countries, households exposed to income loss may cope with food affordability challenges caused by the COVID-19 pandemic mainly by adjusting food consumption rather than using savings [6]. Especially, consumers could shift from relatively expensive but nutrient-rich animal-source foods to relatively cheaper plant-based foods such as cereal and processed foods to cope with income loss [26]. Therefore, animal-source foods consumption is more likely to suffer a reduction during household income loss in developing countries. Even in a rich country such as Germany, meat consumption in those households with income loss has declined since the outbreak of COVID-19 [27]. However, some studies have shown that the consumption of animal-source foods has not been largely affected by the COVID-19 outbreak. Although consumption in almost all food categories was affected by the COVID-19 pandemic in Denmark, Germany, and Slovenia, income loss did not significantly affect the consumption of animal-source foods such as fresh meat, fresh fish, and dairy products [13], while another study also observed that animal-source foods consumption on average remained unchanged in the period between January and August 2020 in Ethiopia [28]. Household savings, borrowing, and social protections such as food assistance or pandemic relief could have played a role in buffering income shock [9,29,30]. Those households with savings had a significantly lower risk of reducing meat, poultry, and vegetable consumption than those without savings during the COVID-19 pandemic [8].

Generally speaking, relatively few studies have examined the immediate impact of household income loss on different food categories, for instance, within a short period of time such as one month. Some studies examining the impact of COVID-19 on food security were conducted by questionnaire. Most surveys were conducted in the second half of 2020 or later, for instance, in October 2020 in African countries [6], and in July 2020 in UAS [12]. Only a few surveys were conducted in the first half of 2020 [13,27]. As far as we know, there have been few empirical studies on the early impact on household consumption of animal-source foods caused by COVID-19 and its containment measures. Although income loss is certainly associated with a decrease in food security for low- and middle-income households over a relatively long period of time, such as a season or longer, the immediate impact of income loss on food consumption remains generally unknown or cannot be agreed upon. Furthermore, under the influence of traditional Chinese culture, Chinese people prefer to save their income for a “rainy day”, compared with some countries’ enthusiasm for consumption in advance. China has one of the highest national savings rates in the world [31]. According to the data released by the world bank, China’s savings accounted for 45.18% of GDP in 2020, far higher than the world average of 29.09% [32]. The high savings rate is an important feature of China’s economic structure and supports China’s rapid economic growth [33]. Residents used savings for consumption to mitigate the impact of COVID-19 on income and daily life [34]. Therefore, the negative impact of household income loss on food consumption in China during the early period of the COVID-19 pandemic could have been offset by the positive effect of household savings on ensuring food security. However, there have been no empirical studies to examine the early influence of household income loss on food security. To bridge this research gap, this study took Nanjing and Wuhan as cases and conducted an online questionnaire survey in March 2020. A binary logistic model was used to analyze the impact of household income loss quantitatively on the consumption of different kinds of animal-source foods during the pandemic. This paper mainly aims to address three research questions: during the early stage of the COVID-19 pandemic in 2020, did household income loss affect household animal-source foods consumption in Nanjing and Wuhan? If so, were there differences in consumption among different types of animal-source foods? What accounts for this difference?

The rest of this paper is structured as follows. Section 2 describes the study area, data source, variable selection, and model specification of this paper. Section 3 provides the results of the empirical study. Section 4 discusses the impact of household income loss and other potential influencing factors on household animal-source foods consumption in Nanjing and Wuhan during the COVID-19 pandemic and the limitations of this study. Section 5 draws conclusions and discusses policy implications.

## 2. Materials and Methods

### 2.1. Study Area

Wuhan and Nanjing were selected as study cases. Wuhan is the capital city of Hubei Province, located in central China [35]. Wuhan implemented lockdown measures, with complete confinement of residential neighborhoods between 14 February 2020 and 8 April 2020, requiring residents to stay at home during that time [15]. The policy of “neighborhood group buying” was developed and implemented. Foods were ordered online or by social medial apps such as WeChat, and then delivered to the neighborhood and distributed to households by people chosen by residential committees [15]. As many business operations stopped during the lockdown period, some households experienced income loss [15].

Nanjing is the capital city of Jiangsu Province and is located in eastern China, around 300 km away from Shanghai. Unlike Wuhan, Nanjing implemented the policy of partial confinement of residential neighborhoods except those neighborhoods with identified cases. Residents were permitted to leave their residential neighborhood to work and buy food and then would have to take body temperature checks when they reentered their neighborhood. The Nanjing Municipal Government made an effort to resume the operation of public food markets, which are the major food outlets for urban residents. Most cities acted as Nanjing did during the pandemic period in 2020, implementing partial closures of residential neighborhoods rather than complete lockdowns.

### 2.2. Data Collection

An online questionnaire survey was conducted in March 2020 to collect data. This study has been reviewed and received ethics clearance through a Wilfrid Laurier University Research Ethics Board (REB#4462), and all participants provided informed consent. When filling out the online questionnaire, people were told that their participation in this study was voluntary. In answering the survey, people could decline to answer any questions they did not wish to answer. People could withdraw their consent at any time without penalty by advising the researcher. All information provided will be used for academic purposes only. Respondents will not be individually identified in any thesis, report or publication resulting from this study. All data will be presented in aggregate form only. The questionnaire was compiled based on the Wenjuanxing platform (Ranxing Information Technology Co., Ltd., Changsha, China), a widely-used e-questionnaire platform in China. The survey questionnaire was distributed via WeChat, a popular social media app in China. Restrictions were placed on access to questionnaires through respondents’ IP addresses to ensure respondents were located in Wuhan or Nanjing during the survey period [36]. If the questionnaire was completed in less than 150 s, or data on key variables were missing, these samples were excluded. Ultimately, there were 1301 completed surveys used for analysis in this study, with 817 from Nanjing and 484 from Wuhan.

### 2.3. Dependent and Independent Variables

#### 2.3.1. Dependent Variable

Food items were grouped as 12 categories in the questionnaire survey. The 12 categories included cereal, roots or tubers, vegetables, fruits, meats, eggs, fish or shellfish, beans or nuts, milk, oil, sugar, and condiments. The animal-source foods in this study included pork, beef and mutton, poultry meat, aquatic products (fish, dried fish, shellfish, and other aquatic products), eggs and dairy products (milk, yogurt, cheese, and other dairy products). The respondents were asked a series of questions about whether their consumption of the food item was affected since the outbreak of the COVID-19 pandemic. Whether the consumption of the food item was affected here refers to whether the quantity, variety, or quality of consumption was decreased, which was specifically manifested in the restrictions on going out, restrictions on online shopping, insufficient supply (insufficient quantity or variety) of supermarkets or online stores, rising food prices, the insufficient freshness of food, and the impact of the pandemic on household income, etc. A series of binary variables in the model were used as dependent variables. The variable *ASF* was used to represent whether animal-source foods consumption (*ASF*) was affected, with a value of 1 if any kind of animal-source consumption was affected, and 0 for not affected. Furthermore, this paper specifically examines the impact of income loss on animal-source foods items such as pork, beef and mutton, poultry meat, aquatic products, eggs, and dairy products. Accordingly, a series of dependent variables represent whether consumption of pork, beef and mutton, poultry, aquatic products, eggs, and dairy products was affected during the early stage of the COVID-19 pandemic (Table 1).

#### 2.3.2. Independent Variables

##### Explanatory Variable

The variable *HIL* is a dichotomic variable, and represents whether a household encountered income loss (*HIL*) and is used as the explanatory variable in this study. If household income loss occurred in the early stages of the COVID-19 pandemic, the variable *HIL* is coded as 1, and 0 for otherwise. Household income loss is an important economic factor affecting animal-source foods consumption [37,38,39]. A study conducted in Tanzania demonstrates that the demand for livestock products (such as meat, milk, and eggs) is expected to increase as income increases [40]. Generally speaking, the increase in the income of urban and rural residents results in the growth of animal products consumption [41,42]. On the contrary, household income loss leads to a decrease in a household’s ability to pay for foods. For these households who suffer income loss, the quantity, variety, or quality of their consumption of animal-source foods declines. The consumption of those relatively expensive food items declines to ensure the ability to meet necessary food intake for adequate nourishment. Therefore, it is hypothesized that the loss of household income will lead to a decline in the quantity, variety, or quality of animal-source foods consumption.

##### Control Variables

Food affordability is mainly affected by household income and food prices [43], among which food prices are also an important factor affecting animal-source foods consumption [44,45,46]. According to the survey results, food price changes are categorized as no rise, a 1–2-fold rise and more than a 2-fold rise, and set as two dummy variables: *HFP* and *MHFP*. The variable *HFP* is used to represent whether food prices are higher than before but less than twice as high as before. The variable *MHFP* is used to represent whether food prices are more than twice as high as before. According to the United Nations World Food Programme (WFP), the COVID-19 pandemic has caused global food prices to soar [47]. Normally, the demand for a commodity is inversely related to its price. Therefore, it is predicted that rising food prices will reduce animal-source foods consumption. Therefore, the variables *HFP* and *MHFP* are hypothesized to have positive coefficients.

Household head gender and age could influence diet structure [48,49]. Gender affects food consumption—“not only women as individuals but also women as head of household” (2016: p1) [50]. The variable *GENDER* is used to represent whether the respondent is female, as a proxy for household head gender. Compared with men, women tend to eat more vegetables and fruits, and consume fewer animal-source foods for nutritional health and weight loss [51]. Therefore, the variable *GENDER* is hypothesized to have negative coefficients. Residents of different ages have different food needs and preferences, which will affect the consumption of animal-source foods [52]. According to the survey results, the age of the respondents, as a proxy for household head age, is divided into three segments: 0–35, 36–59, 60–92, and set as two dummy variables: *MA*, *OA*. The variable *MA* is used to represent whether the respondent is between the ages of 36 and 59 years. The variable *OA* is used to represent whether the respondent is between the ages of 60 and 92 years. According to the consumption trends of Chinese residents, young people tend to consume dairy and animal products, and middle-aged people tend to consume poultry [53]. Older people tend to consume more vegetables and fruits and fewer animal and poultry products [48]. Therefore, the variables *MA* and *OA* are hypothesized to have negative coefficients.

Household structure is an important factor influencing household food consumption and food security [54,55]. These variables are used to capture the impact of household structure on animal-source foods consumption. The variable *FS* represents household structure and refers to whether a household is an extended household, which means a household with husband and wife, children, and relatives of husband and/or wife. Besides the variable *FS*, the variable *PWI* is also used to measure household structure, which refers to whether respondents live with pregnant women/infants. Compared with other types of household structures, extended households have more household members. One study has shown that, the larger the family size, the smaller the per capita consumption of animal-source foods, but the total consumption of animal-source foods will increase accordingly [56]. In addition, those households with seniors or children are more likely to buy beef [31]. Studies show that whether respondents live with pregnant women/infants also affects household animal-source foods consumption [57]. Households living with pregnant women/infants have higher Engel coefficients [58], and those households will spend more money meeting the consumption of animal-source foods for pregnant women/infants to offset some of the negative impact of household income loss. Therefore, the variable *FS* (household structure) is hypothesized to have a positive coefficient, while the variable *PWI* is hypothesized to have a negative coefficient.

Studies have shown that there is a correlation between household housing tenure and household animal-source foods consumption [59]. This study categorizes residents’ housing as owned or leased, and the dummy variable *LEASE* is used to represent whether the housing tenure is leased. A study shows that, when housing tenure is owned, households tend to reduce per capita consumption [59]. Compared with households whose housing tenure is leased, households that own their housing spend more on housing, resulting in less consumption of other goods such as animal-source foods, which is an obvious crowding-out effect [59]. In contrast, the household animal-source foods consumption with leased housing tenure is more, so it is more affected by household income loss. Therefore, the variable *LEASE* is hypothesized to have a positive coefficient.

Lockdown measures had an impact on household animal-source foods consumption [60]. The variable *CC* is used to represent whether the residence was under a completely closed lockdown. Studies have shown that lockdown measures during the COVID-19 pandemic hindered residents’ access to animal-source foods [60]. Therefore, it is speculated that the completely closed management of residences has a negative impact on animal-source foods consumption. The variable *CC* is hypothesized to have a positive coefficient.

Studies have shown that household animal-source foods consumption is associated with location of the residential city of the respondent [61]. Therefore, the variable *CITY* is used to represent whether the residential city of the respondent is Nanjing or Wuhan. If the residential city of the respondent is Nanjing, the variable *CITY* is coded as 1, and 0 for Wuhan. There are differences between the natural environment, economic environment, and social environment in different regions, resulting in significant differences in the consumption preferences of animal-source foods [62,63]. In addition, consumption levels vary widely by regions meaning that the consumption structure between regions is also different [64]. Therefore, the variable *CITY* is hypothesized to have a significant impact on animal-source foods consumption.

Table 2 presents the definitions of the independent variables. The data obtained from the questionnaire are cross-sectional data, from which it is possible to select whether the pandemic affects household income, age, gender, city, household structure, food prices, housing tenure, and lockdown measures. Since the outbreak, whether respondents live with pregnant women/infants and lockdown measures are used as explanatory variables (Table 2). Age, food prices, housing tenure, and lockdown measures are set as dummy variables for later qualitative analysis.

### 2.4. Regression Analysis and Model Specification

There are seven dependent variables in this study (Table 1), which are used to reflect whether animal-source foods consumption, measured through six kinds of animal food consumption, were affected. The six kinds of animal foods are pork, beef and mutton, poultry meat, aquatic products, eggs, and dairy products, which are the main animal-sourced foods consumed in China. As the seven dependent variables are binary variables, it is reasonable to use the binomial logistic regression model for those binary variables [65].

The basic form of the logistic regression model:

(1) $$p_{i}=F\left(y\right)=\frac{e^{\alpha +\sum_{i=1}^{n}\beta_{i}X_{i}}}{1+e^{\alpha +\sum_{i=1}^{n}\beta_{i}X_{i}}}$$

Logistic transformation is performed on Equation (1) to obtain the linear regression model between the probability function and independent variables:

(2) $$ln\frac{p_{i}}{1-p_{i}}=\alpha +\sum_{i=1}^{n}\beta_{i}X_{i}$$

In Equations (1) and (2), *p_i_* represents the probability that animal-source foods consumption is affected; *y* is the dependent variable, indicating whether animal-source-foods consumption is affected, affected = 1, unaffected = 0; *X_i_* is the independent variable, and represents the *i* factor influencing animal-sources food; and *B_i_* is the vector of coefficient of the independent variables.

## 3. Results

### 3.1. Animal-Sourced Foods Consumption during the Early Period of the COVID-19 Pandemic

**Figure 1:**
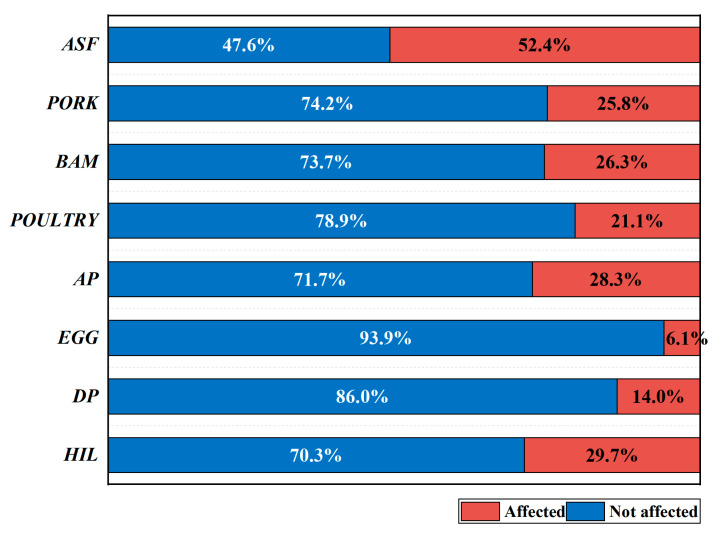
The impact on consumption of animal-source foods and household income. Note: *ASF* = consumption of animal-source foods; *PORK* = consumption of pork; *BAM* = consumption of beef and mutton; *POULTRY* = consumption of poultry; *AP* = consumption of aquatic products; *EGG* = consumption of eggs; *DP* = consumption of dairy products; *HIL* = household income.

There was an extensive adverse impact on the consumption of animal foods during the early stage of the COVID-19 pandemic in 2020, while there were variations in the degree to which different food items were affected. A total of 52.4% of respondents reported that their consumption of animal-source foods was affected during the early period of the COVID-19 pandemic in 2020, which suggests that about half of the households’ animal-source foods consumption was affected by the pandemic. At the same time, there are some differences in the insecurity of different kinds of animal-source foods. Figure 1 shows details of whether various animal-source foods and household income were affected by the COVID-19 pandemic. About 25.8% of families were affected by the pandemic in obtaining pork, 26.3% of families were affected by the pandemic in obtaining beef and mutton, 21.1% of families were affected by the pandemic in obtaining poultry meat, 28.3% of families were affected by the pandemic in obtaining aquatic products, 6.1% of families were affected by the pandemic in obtaining eggs, and 14.0% of families were affected by the pandemic in obtaining dairy products. In addition, 29.7% of households experienced a decline in income due to the pandemic.

There was a remarkable difference in the proportions of animal-source foods consumption being affected between Nanjing and Wuhan. The impact of the pandemic on Wuhan and Nanjing was different, and the proportion of households whose animal-source foods consumption was affected or whose income suffered in the total number of households surveyed in Wuhan and Nanjing was also different. It can be seen from Table 3 that Wuhan was seriously affected by the pandemic, and the number of households whose animal-source foods consumption was affected or whose income suffered is relatively large. Furthermore, the proportion of households whose animal-source foods consumption was affected in Wuhan is 27.4% higher than in Nanjing. The proportion of households whose pork consumption was affected in Wuhan is 14.2% higher than in Nanjing. The proportion of households whose beef and mutton consumption was affected in Wuhan is 26.5% higher than in Nanjing. The proportion of households whose poultry consumption was affected in Wuhan is 15.5% higher than in Nanjing. The proportion of households whose aquatic product consumption was affected in Wuhan is 24.8% higher than in Nanjing. The proportion of households whose egg consumption was affected in Wuhan is 3.2% higher than in Nanjing. The proportion of households whose dairy product consumption was affected in Wuhan is 16.2% higher than in Nanjing. It can be seen that egg consumption was the least affected gap between Wuhan and Nanjing during the early stage of the COVID-19 pandemic. The pandemic caused a serious loss to household income in Wuhan, with 47.7% of the surveyed households there suffering income loss. The difference in the proportion of households suffering from income loss between Wuhan and Nanjing is as high as 28.8%, which indicates that the range of household income loss caused by the pandemic in Wuhan is much larger than that in Nanjing.

### 3.2. Estimation Results

The backward stepwise regression method was used to estimate the seven models with *ASF*, *PORK*, *BAM*, *POULTRY*, *AP*, *EGG*, and *DP* as the dependent variables. The estimation results are shown in Table 4. Logistic regression models are sensitive to multicollinearity among independent variables. Therefore, multicollinearity among variables should be tested [66]. It is generally believed that a tolerance of less than 0.2 is a sign of the existence of multicollinearity, and less than 0.1 indicates that multicollinearity is very serious [67]. The estimation results indicate that there is no multicollinearity in these seven sets of results. On this basis, the Hosmer–Lemeshow test was used to check the goodness of fit of these seven groups of models. It is generally believed that when the *p*-value of the HL test is greater than 0.05, the model fits the data well, and there is no significant difference between the observed data and the predicted data [68]. The *p*-value of the Hosmer–Lemeshow test in the seven groups of results is greater than 0.05, indicating that the goodness of fit of these seven groups of models is good.

The results of logistic regression show that the consumption of most animal-source foods was affected by the variables *HIL* (household income loss), *MHFP* (higher food prices, and more than twice as before), and *CITY* (the residential city of the respondent).

Above all, the variable *HIL* had a significant impact on animal-source foods and the six kinds of animal-source foods, and the symbols are consistent with the expected results. For households suffering income losses, the odds of animal-source foods consumption being affected were increased by a factor of 1.802–2.835, holding other variables constant. It can be seen that household income loss leads to a significant increase in the odds of animal-source foods consumption being affected.

The variable *MHFP* had a significant impact on animal-source foods, pork, aquatic products, and dairy products, and the symbols are consistent with the expected results. For higher food prices, and more than twice as before, the odds of animal-source foods consumption being affected were increased by a factor of 1.336–1.907, holding other variables constant. The odds ratios show, that for double the price, the consumption of aquatic products and pork was the least affected, and dairy products consumption was the most affected. When the price of certain animal-source foods rises, residents reduce such animal-source foods consumption and increase the consumption of foods that can replace such animal-source foods, although it is difficult to replace some animal-source foods, such as aquatic products, because of their high nutritional value,. Nutrients contained in dairy products can also be obtained from meat, eggs, bean products and green vegetables. Therefore, when prices of dairy products rise, people will buy other foods that can replace their nutritional value, thus reducing dairy consumption.

The variable *CITY* had a significant impact on animal-source foods, pork, beef and mutton, poultry, aquatic products, and dairy products, and the symbols are consistent with the expected results. For Nanjing households, the odds of animal-source foods consumption not being affected were increased by a factor of 0.317–0.635, holding other variables constant. Nanjing and Wuhan are both in southern China, and there is little difference in food consumption. Although the government provided material support, the animal-source foods consumption in Wuhan was greatly affected by the pandemic during the study period. The odds ratios show that households in Nanjing consumed more pork, poultry, and dairy products than aquatic products, and beef and mutton.

However, some factors only had a significant impact on the consumption of a few animal-source foods, such as *HFP* (higher food prices, but less than twice as before), *GENDER* (gender of the respondent), *MA* (middle-aged), *OA* (old-aged), *FS* (extended household), *PWI* (living with pregnant women/infants), *LEASE* (lease housing tenure), and *CC* (completely closed management of residence).

Among them, the variable *HFP* (higher food prices, but less than twice as before) had a significant impact on animal-source foods and pork, and the symbols are consistent with the expected results. For higher food prices, but less than twice as before, the odds of animal-source foods consumption being affected were increased by a factor of 1.327–1.348, holding other variables constant.

The variable *GENDER* (gender of the respondent) had a significant impact on pork, beef and mutton, and eggs, and the symbols are as expected. According to the odds ratios, for female respondents, the odds of animal-source foods consumption not being affected were increased by a factor of 0.580–0.772, holding other variables constant. Generally speaking, women consume more vegetables and fruits and less animal-source foods for nutrition, health, and weight loss, leading to less impact on animal-source foods consumption than men during the pandemic. The odds ratios show that women consumed more pork and eggs than beef and mutton. The variable of *MA* (middle-aged) had a significant impact on animal-source foods, pork, and poultry, and the symbols are consistent with the expected results. For middle-aged respondents, the odds of animal-source foods consumption not being affected were increased by a factor of 0.766–0.787, holding other variables constant. The variable of *OA* (old-aged) had a significant impact on animal-source foods, beef and mutton, and poultry, and the symbols are consistent with the expected results. For old-aged respondents, the odds of animal-source foods consumption not being affected were increased by a factor of 0.150–0.445, holding other variables constant. Compared with young people, the middle-aged and old-aged pay more attention to the impact of food consumption on nutritional status and physical health, leading to consuming more vegetables, fruits, etc., and less livestock products [48]. The odds ratios show that the middle-aged consumed more poultry than pork, and the old-aged consumed more poultry than beef and mutton.

The variable *FS* (extended household) had a significant impact on animal-source foods and pork, and the symbols are consistent with the expected results. For extended households, the odds of animal-source foods consumption being affected were increased by a factor of 1.249–1.446, holding other variables constant. The variable *PWI* (living with pregnant women/infants) had a significant impact on animal-source foods and pork, and the symbols are consistent with the expected results. For households living with pregnant women/infants, the odds of animal-source foods consumption not being affected were increased by a factor of 0.734–0.738, holding other variables constant. Households with pregnant women/infants tended to have higher Engel coefficients, and households tended to spend more on animal-sourced foods consumption to ensure adequate nutrition for pregnant women/infants [69].

The variable *LEASE* (lease housing tenure) had a significant impact on animal-source foods, pork, and dairy products and the symbols are consistent with the expected results. For households whose housing tenure was leased, the odds of animal-source foods consumption being affected were increased by a factor of 1.419–1.695, holding other variables constant. Households with leased housing tenure had a higher per capita consumption level [56] and were more vulnerable to the pandemic. The odds ratios show that households with leased housing tenure consumed more pork, and fewer dairy products.

The variable *CC* (completely closed management of residence) had a significant impact on dairy products, and the symbol is consistent with the expected result. Within the completely closed management of residence, the odds of dairy products consumption being affected were increased by a factor of 1.761, holding other variables constant. When lockdown measures were taken, residents were more inclined to use their income to obtain staple food and other rigid needs, thus reducing animal-source foods consumption such as dairy products.

## 4. Discussion

### 4.1. Household Income Loss and Consumption of Animal-Source Foods

Income is the determinant of consumption, which has been confirmed in many traditional economic theories. Among them, the absolute income hypothesis believes that the absolute level of income determines consumption [70], the relative income hypothesis believes that the distribution of income and the highest income level in consumer history determine consumption [71], and the permanent income hypothesis believes that the level of permanent income determines consumption [72]. It can be seen that income is an important factor in determining consumption. Among them, resident income can be divided into wage income, transfer income, operating income, and property income [73]. The outbreak of COVID-19 has had a great impact on residents’ income. According to the data released in the first quarter of 2020, the per capita disposable income of Chinese residents decreased significantly, and the impact of COVID-19 on residents’ wage income was particularly significant [74]. In addition, Bennett’s Law points out that an increase in income leads to the diversification of residents’ diets, a decrease in the consumption of grains and potatoes, and an increase in the consumption of meat, eggs, milk, etc. [75]. From the beginning of reform and opening up to the present, along with economic development and income growth, the overall change of consumption structure is in line with Bennett’s Law [75]. Therefore, the consumption of animal-source foods decreases with the decline of residents’ income. Moreover, the decline in income level not only affects residents’ food consumption, but also cause changes in food consumption structure. The regression analysis results in Table 4 suggest that the decline in income level leads to the reduction in residents’ consumption of animal-source foods, and the impact on various animal-source foods consumption is different, causing changes in the food consumption structure. Some theories revise the income determination theory and add other factors that may affect household consumption, such as food prices and savings, etc. However, in essence, the basis of residents’ choice of animal-source foods consumption is income security. Income has an important impact on changes in demand for food consumption [76]. Food, especially animal-source foods, is a basic necessity, and the consumption of food is undoubtedly greatly affected by household income loss. This is confirmed by the estimation results, which show that household income loss has a significantly negative impact on animal-source foods consumption. The coefficients of the variable *HIL* are statistically significant at 1% level for all seven models (Table 4). The signs for the coefficients of variable *HIL* are consistent with expectations and all are positive, which indicates that experiencing household income loss did increase the likelihood of animal-source foods consumption being affected. According to the odds ratios, for households suffering income losses, the odds of animal-source foods consumption being affected were increased by a factor of 2.203, holding other variables constant. This result may be related to food prices in China, where animal-source foods are usually more expensive than vegetables and fruits, which is borne out by data from the China Yearbook of Agricultural Price Survey 2022. According to the data from the China Yearbook of Agricultural Price Survey 2022, the market prices of animal-source foods (except eggs) are higher than the market prices of vegetables and fruits [77]. Thus, when Chinese households suffer income loss, they are more likely to cut back on these more expensive foods, and buy cheaper ones, such as vegetables and fruits.

Household income loss has different effects on the consumption of various animal-source foods. Dairy products are the most likely to be affected by household income loss. For households suffering income losses, the odds of dairy products consumption being affected increased by a factor of 2.835, holding other variables constant. Eggs consumption was least likely to be affected by household income loss. For households suffering income losses, the odds of eggs consumption being affected were increased by a factor of 1.802, holding other variables constant. In addition, the consumption of other animal-source foods was also affected by household income loss. For households suffering income losses, the odds of pork consumption being affected were increased by a factor of 1.894, holding other variables constant. For households suffering income losses, the odds of beef and mutton consumption being affected were increased by a factor of 2.140, holding other variables constant. For households suffering income losses, the odds of poultry consumption being affected were increased by a factor of 2.773, holding other variables constant. For households suffering income losses, the odds of aquatic products consumption being affected were increased by a factor of 2.345, holding other variables constant. For households suffering income losses, the odds of various animal-source foods consumption being affected were increased by a factor of 1.802–2.835, holding other variables constant. Thus, animal-source foods consumption by households whose income was not guaranteed during the pandemic was greatly affected compared with households whose income was guaranteed, indicating that household income is an important factor in animal-source foods consumption. Moreover, the results indicate that household income loss affected various animal-source foods consumption differently. Among them, the demand for poultry and dairy products fluctuated greatly with income, indicating that the consumption demand of residents in Nanjing and Wuhan for poultry and dairy products decreased during the pandemic, and the residents paid less attention to poultry and dairy products consumption, which may be related to the consumption habits and dietary structure of Chinese residents. Pork and aquatic products occupy the major position in the average annual consumption of animal-source foods of Chinese residents [78]. Therefore, for the residents of Nanjing or Wuhan, pork and aquatic products possess the characteristics of daily necessities [79]. When faced with household income loss, pork and aquatic products may have been less affected than other types of animal-source foods. However, the empirical results that the consumption of aquatic products has been greatly affected are not completely consistent with this speculation. This may be caused by the samples selected in this paper being from Wuhan and Nanjing, both of which are not coastal cities, so the self-sufficiency of aquatic products could not be guaranteed, and the traffic disruption caused by the pandemic may have caused great difficulties in the transportation of aquatic products, which may have led to a greater impact on the consumption of aquatic products. The consumption of poultry, dairy products, and beef and mutton accounts for a relatively small proportion of the consumption of animal-source foods by Chinese residents [78]. Therefore, in the face of household income loss, Chinese residents are more likely to reduce their consumption of these kinds of animal-source foods, and more likely to consume foods that conform to their dietary habits, such as pork. Although eggs also account for a relatively small proportion of the consumption of animal-source foods by Chinese residents [78], China is a major producer of eggs, and eggs are the cheapest among animal-source foods [80]. Therefore, eggs consumption may be less affected in the face of household income loss.

### 4.2. Immediate Impact and Combination of Price Increase, Income Loss and Insufficient Savings

#### 4.2.1. Immediate Impact Associated with a Combination of Three Adverse Factors

**Figure 2:**
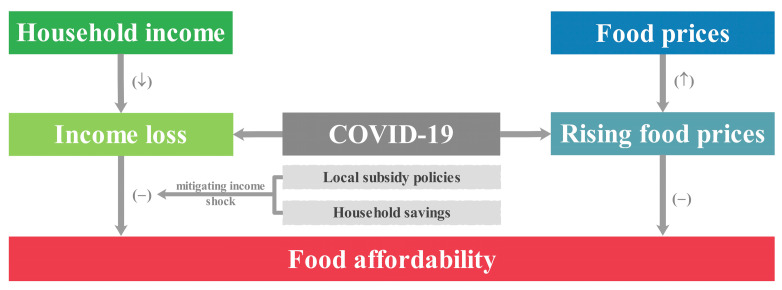
Combination of potential adverse factors changing food affordability during an emergency.

Income and food prices are two proximate factors determining food affordability [81]. When talking about food affordability in an emergency context, household savings, and subsidy and food remittance are also important, along with household income and food prices (Figure 2). The negative effects of emergencies such as the COVID-19 pandemic are often not unilateral, but simultaneously multiple, such as income loss, price increase, and insufficient savings. The combination of three adverse factors created a “perfect storm” for animal-source foods consumption during the early stage of the COVID-19 pandemic [82]. It also indicates that household income loss had an immediate impact on animal-source foods consumption during the early stage of the COVID-19 pandemic. Household income is an important factor changing food affordability, as demonstrated in the research in urban Zambia and Kenya, where rising real formal sector wages contributed to increasing staple food affordability in both countries [83]. A study shows that the food affordability for households improved over this period due to an increase of average weekly earnings and welfare payments [43]. Food price fluctuations lead to high variability in food affordability in repeated pandemic shocks [84]. The reduction of the price of the average diet, corresponding to an increase in its affordability, is due to the decrease in consumption of expensive commodities (such as animal products), which is then compensated for with an increased consumption of fruits and vegetables [84]. The consumption level drops due to the income loss caused by the pandemic. To avoid a sharp drop in future consumption levels caused by uncertain factors, residents should make precautionary savings in advance [34], as this is one of the important factors for changing food affordability. A study showed that households using savings lowered the hazard of food insecurity, compared with those households having to borrow money during the COVID-19 pandemic [85]. Inequity in the affordability of healthy food is a major public health concern and one that demands recognition and national action. The impact of local subsidy policies affecting welfare support and wages needs to be considered, as well as food pricing strategies and possible food subsidies for those at greatest risk of food insecurity [43].

#### 4.2.2. Food Prices Fluctuations, Household Income Loss and Animal Foods Consumption

**Figure 3:**
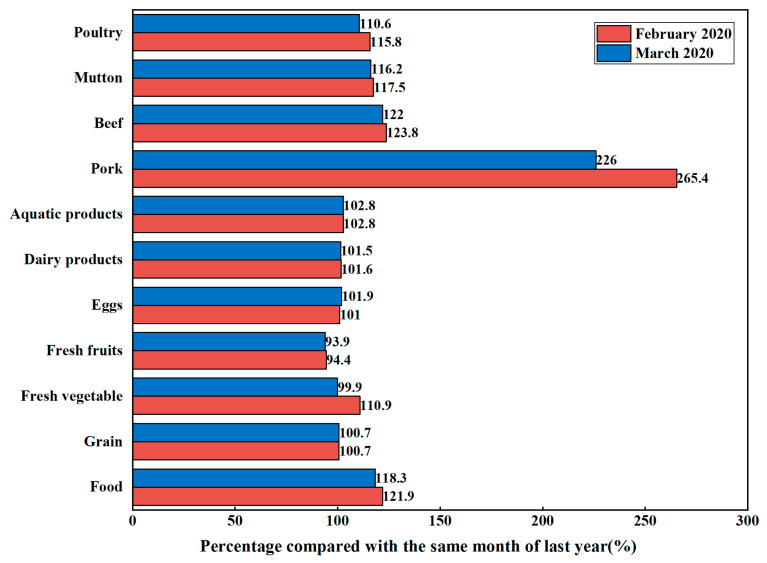
Percentage compared with the same month of the previous year (same month of previous year = 100%). Note: The values of food, grains, fresh vegetables, fresh fruits, eggs, and aquatic products are the percentage of consumer price index compared with the same month of the previous year. The value of dairy products is the percentage of producer price index compared with the same month of the previous year. The values of pork, beef, mutton, and poultry are the percentage of market price compared with the same month of the previous year. The data in the figure are from the National Bureau of Statistics.

The spread of the COVID-19 pandemic has brought great challenges to the supply, transportation, and sales of agricultural products, resulting in increased transportation costs and higher food prices. In February 2020, the consumer price index, producer price index, and market price were at an all-time high. All values except the value of fresh fruits were higher than in the same month of the previous year (Figure 3). According to the data released by the National Bureau of Statistics: from a year-on-year perspective, in March 2020, the national consumer price rose by 4.3% year-on-year. Among them, urban rose by 4.0%, rural rose by 5.3%, and food prices rose by 18.3% [86]. In March, the number of seasonal vegetables in the spring increased, and the prices of many foods, such as fresh vegetables and fruits, declined significantly. The consumer price indices of fresh fruits and fresh vegetables in March were lower than those in the same month of the previous year, but the consumer price indices of eggs and aquatic products, the producer price index of dairy products, and the market prices of pork, beef, mutton, and poultry still showed an upward trend from a year-on-year perspective, as shown in Figure 3. Households suffering from income loss may have been more inclined to consume foods such as fresh vegetables at lower prices than to buy animal-source foods at higher prices. Due to the impact of the COVID-19 pandemic, the prices of animal-source foods have increased, especially the price of pork, which has increased the burden of purchasing animal-source foods for those with income loss.

#### 4.2.3. Household Savings, Income Loss and Animal Foods Consumption

To avoid a sharp drop in future consumption levels caused by uncertain factors, residents should make precautionary savings in advance [34]. When the consumption level drops due to the income loss caused by the pandemic, the food value chain can resist some COVID-19-related shocks [28]. A study showed that using savings lowered the risk of food insecurity, compared with those households that had to borrow money during the COVID-19 pandemic [85]. Having no savings to buy food generally increases the risk of household food insecurity [87]. In China, savings and residents’ savings deposits generally refer to individual accounts in banks [88], including current savings accounts, time savings accounts, and other savings accounts [89]. The savings of residents in Nanjing and Wuhan reduced the impact of income loss caused by the pandemic on residents’ animal-source foods consumption to a certain extent. However, according to the China Household Finance Survey Report (2012), 55% of households have no or few savings [90] to help them withstand a drop in consumption from income loss. According to the China Household Finance Survey Report (2014), only 56.6% of the national effective samples have a current savings account, and only 17.4% have a time savings account. Moreover, the distribution of household savings in China is extremely uneven [91]. Therefore, the results show that animal-source foods consumption was still negatively affected by income loss on the whole.

#### 4.2.4. Local Subsidy Policies, Household Income Loss and Animal-Source Foods Consumption

Despite a series of measures taken by the government during the early stage of the COVID-19 pandemic, household income was still seriously affected by the pandemic, with 29.6% of households suffering income losses, indicating that local subsidy policies did not respond to the impact of the pandemic on household income in a timely enough manner during the early stage of the COVID-19 pandemic. On 10 February 2020, Wuhan issued a notice on actively responding to pandemic prevention and control, and supporting the development of enterprises with adequate social insurance policies [92], proposing to implement the rate reduction policy, increase the return of stable posts, and increase employment subsidies, etc. On 19 February 2020, Nanjing issued a notice on printing and distributing several policies and measures to deal with the COVID-19 pandemic and ensure stable employment [93], proposing to implement the unemployment insurance return policy and provide employment subsidies. These policy documents played a certain role in alleviating the income loss of employees, thus further alleviating the negative impact of the loss of household income on animal-source foods consumption, strengthening the protection of food consumption for the groups suffering from income loss, and making emergency plans in advance for future public health incidents and other public emergencies.

### 4.3. Research Limitations

Given the risk of COVID-19 and the lockdown, we were unable to enter Wuhan or conduct field research in Nanjing. There may be some selection bias and insufficient sample size in the study sample. First, the study used a quick, web-based survey. The online questionnaire could not use probability-based sampling (such as stratified sampling) to identify the households of the respondents, but the samples covered most areas of Wuhan and Nanjing in geographical space, and there was no significant concentration or sparsity. Questionnaires for the study were distributed via WeChat, China’s most popular social media platform. In 2019, the monthly active users of WeChat accounted for 82.143% of the total population in China [94]. In WeChat, households in the same community create groups based on location, while households in different communities create groups based on other relationships, such as work relationships, to connect people of different classes via a virtual network. This study assumed that the spread pattern of questionnaires in WeChat groups was similar to random sampling or snowball sampling in reality. In an earlier study, a similar method was used to investigate household food diversity online during the pandemic [95]. Second, due to the characteristics of online interviews, marginalized groups such as elderly people living alone who did not use social media software regularly were unlikely to be covered. The absence of this marginal group may have affected the results of age-related studies on food consumption of animal-source foods. Third, similar to other studies that use online surveys, the answers to all questions in the questionnaire were self-reported and may deviate from the actual situation. However, the effect could be mitigated to some extent by completely anonymous online surveys. Finally, there may have been some limitations in the content of the online questionnaire. Due to the need to obtain data as soon as possible in the early stage of the COVID-19 pandemic, our online questionnaire content was limited. We did not investigate the reduction in animal-source foods consumption or the impact of reduction in animal-source foods consumption on human health, and we would pay attention to these issues in future research.

## 5. Conclusions and Policy Implications

At the beginning of 2020, a sudden epidemic swept across China. To control the pandemic, China undertook a series of lockdown measures, including strict control of traffic, suspension of production and work, and prohibition of gatherings. These efforts effectively controlled the risk of the outbreak and the spread of the pandemic, but they have also led to household income loss, reduced access to food, and increased food insecurity. This study investigated the consumption of animal-source foods by residents in Nanjing and Wuhan in the early stage of the COVID-19 pandemic, and applied descriptive analysis and econometric methods to investigate the impact of household income loss on animal-source foods consumption in the early stage of the COVID-19 pandemic. This study selected household income loss, food prices, and other variables that may have affected animal-source foods consumption, and conducted empirical analysis to verify the impact of these variables on animal-source foods consumption.

Results of this study indicate that household income loss has a significantly negative effect on animal-source foods consumption. Moreover, household income loss has different effects on the consumption of various animal-source foods. Household income loss is a key factor in animal-source foods consumption, and income security is the basis for residents to consume animal-source foods. The income of Chinese residents mainly depends on wage income or operating income. The pandemic has prolonged the resumption of work and reduced working hours. Most small- and medium-sized enterprises have chosen to cut wages and lay off employees to avoid production suspension, resulting in lower incomes. Based on the findings, this study reached the following policy implications: the government should improve the temporary income subsidy system in response to major pandemics and include those in distress due to public emergencies in the scope of assistance, and provide temporary income subsidies to those in severely affected areas. According to the decline in residents’ incomes, the standard of temporary income subsidies or social security funds for low-income residents in the affected areas should be appropriately increased to ensure a good living standard of residents. Regarding food prices, results of this research indicate that rising food prices have a significant negative effect on household consumption of pork, aquatic products, and dairy products. In addition to the impact of their prices on consumption, other food prices also have an impact on consumption because of the substitutability of different foods. When the price of animal-source foods fluctuates greatly, the benign interaction between consumption and production is disrupted. Based on the findings, we suggest the following policy proposition: based on the law of price and consumption changes of animal-source foods in China, the government should strengthen the macro-control of the animal-source foods market, prevent irrational large price fluctuations, start the price subsidy linkage mechanism in a timely manner, improve the subsidy standard, and ensure the consumption capacity of animal-source foods for residents.

During the COVID-19 pandemic, household incomes fell, which had an immediate impact on animal-source foods consumption. This immediate impact may have been due to the combination of price increases, income loss, and insufficient savings, which led to a “perfect storm” impacting animal-source foods consumption. The development of national nutrition intake and health has entered a state of tension, leading to an increase in food security risk in China. The findings of this research may provide a reliable guide to future policy implication for the rational development of animal-source foods consumption structure and food security. The findings of this research may help to analyze the impact of public health emergencies on residents’ living standards and provide policy references for protecting the basic living standards of vulnerable groups and increasing their ability to resist risks.

## Figures and Tables

**Table 1:** The dependent variables used in this study.

Variables	Definition	Mean	Standard Deviation
**Dependent variables**			
*ASF*	Whether consumption of animal-source foods was affected, *ASF* = 1 for yes, otherwise, *ASF* = 0	0.524	0.500
*PORK*	Whether consumption of pork was affected, *PORK* = 1 for yes, 0 for otherwise	0.258	0.438
*BAM*	Whether consumption of beef and mutton was affected, *BAM* = 1 for yes, 0 for otherwise	0.263	0.440
*POULTRY*	Whether consumption of poultry was affected, *POULTRY* = 1 for yes, 0 for otherwise	0.211	0.408
*AP*	Whether consumption of aquatic products was affected, *AP* = 1 for yes, 0 for otherwise	0.283	0.451
*EGG*	Whether consumption of eggs was affected, *EGG* = 1 for yes, 0 for otherwise	0.061	0.239
*DP*	Whether consumption of dairy products was affected, *DP* = 1 for yes, 0 for otherwise	0.140	0.347

**Table 2:** Definition of independent variables.

Variables	Definition	Mean	Standard Deviation
**Explanatory variable**			
*HIL*	Household income loss, *HIL* = 1 for yes, 0 for otherwise	0.297	0.457
**Control variables**			
*HFP*	Food prices are higher than before, but less than twice as before, *HFP* = 1 for yes, 0 for otherwise	0.489	0.500
*MHFP*	Food prices are more than twice as before, *MHFP* = 1 for yes, 0 for otherwise	0.219	0.414
*GENDER*	Gender of respondent, *GENDER* = 1 for woman, 0 for man	0.552	0.499
*MA*	The respondent is middle-aged, *MA* = 1 for yes, 0 for otherwise	0.336	0.472
*OA*	The respondent is old-aged, *OA* = 1 for yes, 0 for otherwise	0.018	0.135
*FS*	Extended household (husband and wife, children, and relatives of husband and wife), *FS* = 1 for yes, 0 for otherwise	0.321	0.467
*PWI*	Live with pregnant women/infants, *PWI* = 1 for yes, 0 for otherwise	0.293	0.455
*LEASE*	Housing tenure, *LEASE* = 1 for lease, 0 for otherwise	0.156	0.363
*CC*	Completely closed management of residence, *CC* = 1 for yes, 0 for otherwise	0.467	0.499
*CITY*	The residential city of the respondent, *CITY* = 1 for Nanjing, 0 for otherwise	0.628	0.484

**Table 3:** Proportion of households experiencing income losses and challenges in animal food consumption.

Item	Wuhan	Nanjing	Difference
Animal-source foods	69.6%	42.2%	27.4%
Pork	34.7%	20.5%	14.2%
Beef and mutton	42.9%	16.4%	26.5%
Poultry	30.7%	15.2%	15.5%
Aquatic products	43.8%	19.0%	24.8%
Eggs	8.0%	4.8%	3.2%
Dairy products	24.1%	7.9%	16.2%
Household income loss	47.7%	18.9%	28.8%

Sources: The table is made based on the online survey conducted in March 2020.

**Table 4:** Binary logistic regression results and probability ratio results of models.

Variables	*ASF*	*PORK*	*BAM*	*POULTRY*	*AP*	*EGG*	*DP*
*HIL*	0.790 *** (2.203)	0.639 *** (1.894)	0.761 *** (2.140)	1.020 *** (2.773)	0.852 *** (2.345)	0.589 *** (1.802)	1.042 *** (2.835)
*HFP*	0.299 ** (1.348)	0.283 * (1.327)					
*MHFP*	0.645 *** (1.907)	0.485 ** (1.624)			0.290 * (1.336)		0.499 *** (1.647)
*GENDER*		−0.546 *** (0.580)	−0.259 * (0.772)			−0.486 ** (0.615)	
*MA*	−0.240 * (0.787)	−0.267 * (0.766)		−0.261 * (0.771)			
*OA*	−0.809 * (0.445)		−1.078 * (0.340)	−1.897 * (0.150)			
*FS*	0.222 * (1.249)	0.369 ** (1.446)					
*PWI*	−0.310 ** (0.734)	−0.304 ** (0.738)					
*LEASE*	0.350 ** (1.419)	0.528 *** (1.695)					0.471 ** (1.602)
*CC*							0.566 *** (1.761)
*CITY*	−0.891 *** (0.410)	−0.455 *** (0.635)	−1.150 *** (0.317)	−0.656 *** (0.519)	−0.906 *** (0.404)		−0.575 *** (0.563)
*p*-value of the HL test	0.809	0.473	0.775	0.434	1.000	0.995	0.895

Notes: The data in parentheses are odds ratios. *** means 1% level significance, ** for 5% level significance, and * for 10% level significance.

## Data Availability

Not applicable.
